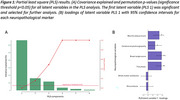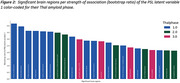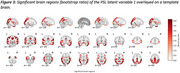# In vivo mean diffusivity is associated with neuropathology markers of Alzheimer's disease

**DOI:** 10.1002/alz70856_106960

**Published:** 2026-01-08

**Authors:** Cécilia Tremblay, Zaki Alasmar, Yashar Zeighami, Mahsa Dadar

**Affiliations:** ^1^ Banner Sun Health Research Institute, Phoenix, AZ, USA; ^2^ Douglas Research Center, Montreal, QC, Canada; ^3^ Mcgill university, Montreal, QC, Canada; ^4^ McGill University, Montreal, QC, Canada; ^5^ Douglas Mental Health University Institute, Montréal, QC, Canada; ^6^ Department of Psychiatry, McGill University, Montréal, QC, Canada; ^7^ McGill University, Montréal, QC, Canada

## Abstract

**Background:**

Diffusion weighted imaging (DWI) of gray matter can identify microstructural changes that have recently been shown to correlate with biomarkers of Alzheimer's disease (AD). Hence DWI‐based metrics such as mean diffusivity (MD) hold great promise to serve as an early and sensitive biomarker of AD pathology (Spotorno et al. 2023, Sun et al. 20). However, its underlying neuropathological determinants remain unclear. We therefore aimed to assess the association between MD and postmortem AD neuropathology.

**Method:**

Cases with in‐vivo DWI within 6 years of death and postmortem neuropathology assessment were obtained from the National Alzheimer's Coordinating Center (NACC) database (*N* = 43). Participant‐specific MD values were extracted from 132 regions of the Allen Anatomical Human Brain Atlas. MD values were harmonized across protocols, and residualized for age at death, sex, and MRI‐to‐death time interval. Partial Least Squares (PLS) analyses were then performed to assess the relationships between regional MD and pathology markers, including Thal amyloid phase, CERAD neuritic plaque score, Braak neurofibrillary stage, cerebral amyloid angiopathy (CAA), white matter rarefaction, and presence of microinfarcts

**Result:**

The PLS analyses revealed one significant latent variable explaining 66% of the shared covariance between MD and neuropathology markers (ppermutation < 0.05). Significant neuropathology measures, in order of strongest contributor, included a higher Braak stage (β=0.57, 95%CI=[0.36,0.74]), Thal phase (β=0.55, 95%CI=[0.35, 0.71]), neuritic plaque score (β=0.47, 95%CI=[0.23, 0.66]) and CAA score (β=0.290572, 95%CI=[0.009, 0.56]). Occipital, parietal, temporal and frontal regions had the strongest association with neuropathology (bootstrap ratio (BSR) > 3.5, *p* <0.05), followed by cingulate cortex, basal forebrain, insula, and hippocampus and amygdala.

**Conclusion:**

These results suggest that MD values, derived from in‐vivo DWI scans, are associated with a combination of neuropathology markers. In other words, the accumulation of both tau and amyloid leading to more severe neuropathology causes microstructural changes that are detectable by non‐invasive in‐vivo DWI biomarkers. This is a first step to validate the use of DWI as a non‐invasive tool to assess the severity of neuropathology in AD.